# Changes in monocyte subsets are associated with clinical outcomes in severe malarial anaemia and cerebral malaria

**DOI:** 10.1038/s41598-019-52579-7

**Published:** 2019-11-26

**Authors:** Jade Royo, Mouna Rahabi, Claire Kamaliddin, Sem Ezinmegnon, David Olagnier, Hélène Authier, Achille Massougbodji, Jules Alao, Yélé Ladipo, Philippe Deloron, Gwladys Bertin, Bernard Pipy, Agnès Coste, Agnès Aubouy

**Affiliations:** 1 PHARMADEV UMR 152, Institut de Recherche pour le Développement (IRD), Université Paul Sabatier Toulouse 3, Toulouse, France; 2 MERIT UMR 216, IRD, Université Paris 5, Sorbonne Paris Cité, Paris, France; 3 Centre d’Etude et de Recherche sur le Paludisme Associé à la Grossesse et l’Enfance (CERPAGE), Cotonou, Benin; 4 Aarhus University, Department of Biomedicine, Research Center for Innate Immunology, Aarhus C, 8000 Denmark; 5 Paediatric Department, Mother and Child University and Hospital Center (CHUMEL), Cotonou, Benin

**Keywords:** Immunology, Biomarkers

## Abstract

Monocytes are plastic heterogeneous immune cells involved in host-parasite interactions critical for malaria pathogenesis. Human monocytes have been subdivided into three populations based on surface expression of CD14 and CD16. We hypothesised that proportions and phenotypes of circulating monocyte subsets can be markers of severity or fatality in children with malaria. To address this question, we compared monocytes sampled in children with uncomplicated malaria, severe malarial anaemia, or cerebral malaria. Flow cytometry was used to distinguish and phenotype monocyte subsets through CD14, CD16, CD36 and TLR2 expression. Data were first analysed by univariate analysis to evaluate their link to severity and death. Second, multinomial logistic regression was used to measure the specific effect of monocyte proportions and phenotypes on severity and death, after adjustments for other variables unrelated to monocytes. Multivariate analysis demonstrated that decreased percentages of non-classical monocytes were associated with death, suggesting that this monocyte subset has a role in resolving malaria. Using univariate analysis, we also showed that the role of non-classical monocytes involves a mostly anti-inflammatory profile and the expression of CD16. Further studies are needed to decipher the functions of this sub-population during severe malaria episodes, and understand the underlying mechanisms.

## Introduction

The two primary severe disease outcomes of malarial infection are cerebral malaria (CM) and severe malarial anaemia (SMA). Case fatality rates among African children with CM remain in the 15–25% range^1–3^ while reported mortality rates for SMA are consistently lower than for CM but vary notwithstanding from 1.4% to 22%^4,5^. Pathophysiology of CM is based on the accumulation of *Plasmodium falciparum*-infected erythrocytes (iE) in the brain and clinically develops into coma^6^. Infected red blood cells adhere to vascular endothelium and other erythrocytes, cause ischemia and hypoxia, elicit endothelial activation and signalling, blood brain barrier impairment and a range of immune cell host responses to resolve the neuroinflammation process^7,8^. Here, monocytes play a central role in secreting immune cytokines, chemokines and reactive oxygen species, participating in immune cell chemoattraction and polarization and in the redox balance^9,10^. SMA is defined by a haemoglobin level of < 5 g/dL. Its aetiology is attributed to the lysis of infected and uninfected red blood cells with anaemia persisting even after successful clearance of parasitemia^11–13^. Dyserythropoiesis and bone marrow suppression explain the low replacement of red blood cells^14–16^. Early Ei elimination by monocyte/macrophages-dependent phagocytosis and reactive oxygen species production drives both the inflammatory response and inhibition of erythropoiesis, while accumulation of hemozoin-containing monocytes has been linked to suppression of erythropoiesis^17^.

Human monocytes are identified by their lipopolysaccharide receptor CD14, a co-receptor for lipopolysaccharide (LPS) which is abundantly expressed. Inflammatory activity of monocytes expressing CD16, a Fcγ receptor, was recognized many years ago^18–20^, which has led to studies demonstrating the heterogeneity of monocytes^21^. Now, human monocytes are divided in three subsets based on both CD14 and CD16 expression, named classical (CD14^+^ CD16^-^), intermediate (CD14^+^ CD16^+^) and non-classical (CD14^low^ CD16^+^). According to their response to LPS stimulation, classical monocytes are known for their major function of phagocytosis with capacity to produce IL-10 and low TNF; intermediate monocytes are known as inflammatory cells with high capacity to produce IL-1β and TNF; and non-classical monocytes are cells patrolling in vessels able to differentiate into anti-inflammatory macrophages during inflammation^22^. However, during pathological processes, the effect of count change remains unclear. We earlier established that proportions and phenotypes of monocyte subsets are related to clinical outcomes in pregnancy-associated malaria^23^. In that study, CD14^low^ CD16^+^ non-classical monocytes increased in comparison to reported proportions of healthy pregnant women, and increased expression of the CD36 scavenger receptor on monocytes was related to higher infant birth weight.

In the current study, we postulated that proportions and phenotypes of circulating monocyte subsets can be a marker of severity or death outcome in children with malaria. To verify this hypothesis, we compared monocytes sampled in children with uncomplicated malaria (UM), SMA or CM. To define monocyte phenotypes, we studied the scavenger receptor CD36 and the Toll-like receptor 2 (TLR2), two pattern recognition receptors known for their functional implication in iE elimination^24–28^. The relationship between monocyte subsets and levels of cytokines, plasma chemokines, and chemokine receptor expression was also investigated. Proportions and phenotypes of monocytes were first analysed by univariate analysis to evaluate their link to severity and death outcome. Second, multinomial logistic regression was used to measure the specific effect of monocyte proportions and phenotypes on severity and death, after adjustment for other variables unrelated to monocytes. Our results highlight the importance of non-classical monocytes in outcomes of severe malaria in children (CM and SMA). Multivariate analysis shows that a decreased percentage of non-classical monocytes is associated with death. Using univariate analysis, we also clarify the relationship of CD14, CD16, CD36 and TLR2 expression by monocyte subsets with severity and death outcomes.

## Results

### Baseline characteristics of patients and relation to severity and death occurrence

A total of 133 patients were enrolled and divided in 42 CM, 29 SMA and 62 UM (Table 1). Blood samples were drawn immediately at inclusion. Not all measures could be assessed in every patient because of early death or low volumes of sampled blood. The characteristics of the patients included in this study are shown in Table 1. Median ages and female/male ratio were similar. Previous hospitalization was more frequent in the UM group (P = 0.001). Axillary temperature was higher in both severe groups (P = 0.0005). In agreement with inclusion criteria, seizures were more frequent and Blantyre score was lower in CM (P < 0.0001). Similarly, haemoglobin was lower in SMA children (*P* = 0.0002 for SMA compared to CM, *P* < 0.0001 for SMA compared to UM by Mann-Whitney U-test). Children presenting with CM were characterized by much higher parasite densities and death occurrence compared to SMA and UM children (*P* < 0.0001 for all comparisons except for death in CM versus SMA, *P* = 0.003 by Mann-Whitney U-test). Plasma IL-1β, IL-6, IL-10, MCP-1 and CXCL10 were also associated with severity. IL-1β and MCP-1 were specifically higher in the CM patients (see Table 1). IL-6, IL-10 and CXCL-10 were similar in CM and SMA patients while these two severe groups presented higher levels than UM patients (see Table 1).

**Table 1:** Baseline characteristics of patients according to clinical groups and univariate analysis of risk factors of malaria severity.

	UM		SMA		CM		*P* value^b,c^
no. of patients	n	62	n	29	n	42	
Age (months)^a,b^	61	30 (18.0–48.0)	27	30.0 (13.7–48.0)	42	32.5 (18.0–48.0)	0.87
Ratio F/M^c^	62	30/32	27	13/14	42	18/24	0.84
Previous hosp. (%)^c^	27	11 (40.7)	26	2 (7.7)	42	4 (9.5)	**0.0012**
Axillary temp.^a,b^	46	37.6 (37.1–38.0)	23	38.0 (37.4–39.0)	41	38.5 (37.9–39.0)	**0.0005**
Seizure (%)^c^	62	0	24	5 (20.8)	42	24 (57.1)	**<0.0001**
Blantyre score^a,b^	62	5 (5–5)	25	5 (4–5)	42	2.0 (1.0–2.0)	**<0.0001**
Glycemia^a,b^	15	0.9 (0.9–1.0)	12	0.9 (0.4–1.2)	36	0.8 (0.3–1.2)	0.92
Hemoglobin^a,b^	21	10.7 (9.6–12.1)	28	4.1 (3.5–4.6)^***,§§§^	41	5.2 (4.1–6.6)	**<0.0001**
Parasite density (/µL of blood)^a,b^	60	2,684 (645–16,588)	29	2,182 (635–34,763)	42	302,534 (15,375–1,346,960)^$$$,§§§^	**<0.0001**
Death (%)^c^	62	0	27	2 (7.4)	37	15 (40.5)^$$$,§§^	**<0.0001**
Plasm. IL-1β^a,d^	54	0 (0–4.7)	28	0 (0–0)	36	33.6 (0–58.4)^$$$,§§§^	**<0.0001**
Plasm. IL-6^a,d^	45	5.3 (0–19.4)	25	38.0 (10.1–185)^***^	36	78.0 (28.0–164)^$$$^	**<0.0001**
Plasm. IL-10^a,d^	61	78.3 (10.1–392)	28	565 (279–2,076)^***^	36	895 (407–1,675)^$$$^	**<0.0001**
Plasm. MCP-1^a,d^	59	150 (96.4–291)	27	150 (78.9–475)	36	234 (141–702)^$,§^	**0.03**
Plasm. CXCL10^a,d^	44	251 (76.6–778)	25	657 (449–857)^*^	36	794 (422–1534)^$$$^	**0.0002**

^a^Median value (interquartile range), ^b^Quantitative variables were compared between groups using the Kruskal-Wallis test, ^c^Categorized variables were compared using a chi-square test, ^d^In pg/mL. Two-by-two comparisons between quantitative variables were tested by the Mann Whitney U-test and indicated by ^*^for UM versus SMA, ^$^for UM versus CM and ^§^for SMA versus CM. One symbol means P < 0.05, two symbols P < 0.005 and 3 symbols P < 0.0005.

Risk factors of malarial death were also studied by univariate analysis of baseline variables (Table 2). Table 2 shows that seizure occurrence, lower Blantyre score, higher parasitaemia and higher plasma IL-1β, IL-6, IL-10 and CXCL-10 are risk factors of death in this study (P < 0.0001, P = 0.008, P = 0.002, P = 0.0008, P = 0.001, P = 0.008, P = 0.002 respectively). Since the death group consists of only 17 patients (12 to 17 according to the parameter) compared to 109 in the survivor group (49 to 108 according to the parameter), the results should be interpreted with caution.

**Table 2:** Univariate analysis of risk factors of malarial death among baseline variables.

	Survival		Death		*P* value^b,c^
no. of patients	n	109	n	17	
Age (months)^a,b^	108	32.0 (18.0–48.0)	15	36.0 (18.7–48.0)	0.62
Ratio F/M^c^	108	50/58	16	10/6	0.23
Previous hosp. (%)^c^	73	16 (21.9)	15	1 (6.7)	0.17
Axillary temp.^a,b^	89	38.0 (37.3–38.8)	14	38.5 (37.8–39.0)	0.08
Seizure (%)^c^	107	19 (17.8)	15	6 (40.0)	**0.05**
Blantyre score^a,b^	108	5 (4–5)	15	1 (1–2)	**<0.0001**
Glycemia^a,b^	49	0.90 (0.67–1.12)	12	0.32 (0.17–1.34)	0.25
Hemoglobin^a,b^	68	5.1 (4.1–9.5)	16	5.0 (3.9–6.9)	0.35
Parasite density (/µL of blood)^a,b^	107	5,533 (841–50,402)	17	40,000 (4,154–1,510,000)	**0.008**
Plasm. IL-1β^a^^,^^d^	97	0 (0–12.1)	14	42.4 (5.4–116)	**0.0008**
Plasm. IL-6^a,d^	85	12.9 (1.3–83.3)	14	103 (42.5–199)	**0.001**
Plasm. IL-10^a,d^	104	341 (38–1125)	14	860 (617–1690)	**0.008**
Plasm. MCP-1^a,d^	101	156 (102–345)	14	325 (146–1,641)	0.06
Plasm. CXCL10^a,d^	84	531 (218–884)	14	1232 (574–1849)	**0.002**

^a^Median value (interquartile range), ^b^Quantitative variables were compared between groups using the Kruskal-Wallis test, ^c^Categorized variables were compared using a chi-square test, ^d^In pg/mL.

### Proportions of monocyte subtypes and relation to malarial severity and death

**Figure 1:**
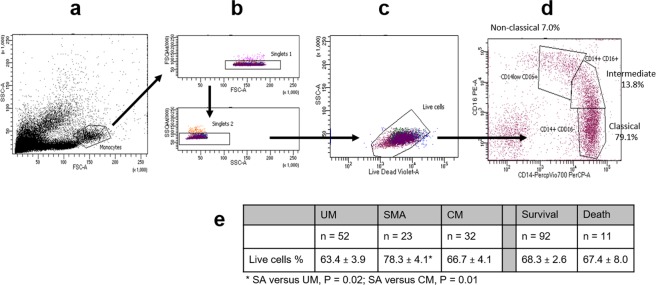
Gating strategy for the identification of monocyte sub-populations by flow cytometry. Forward and side scattering areas (FSC-A and SSC-A) were first used to gate monocytes among other leucocytes (**a**). Doublets were removed by gating on forward-scatter-width (FSC-W) versus FSC-A and side-scatter-width (SSC-W) versus SSC-A (**b**). Dead cells were excluded from the single-cell population (**c**). Monocytes were subdivided into CD14+CD16−/CD14+CD16+/CD14low CD16+, according to CD14 and CD16 staining characteristics (**d**). Percentages of live cells are indicated according to the clinical group or the clinical outcome and values were compared by the Mann-Whitney U-test (**e**).

**Figure 2:**
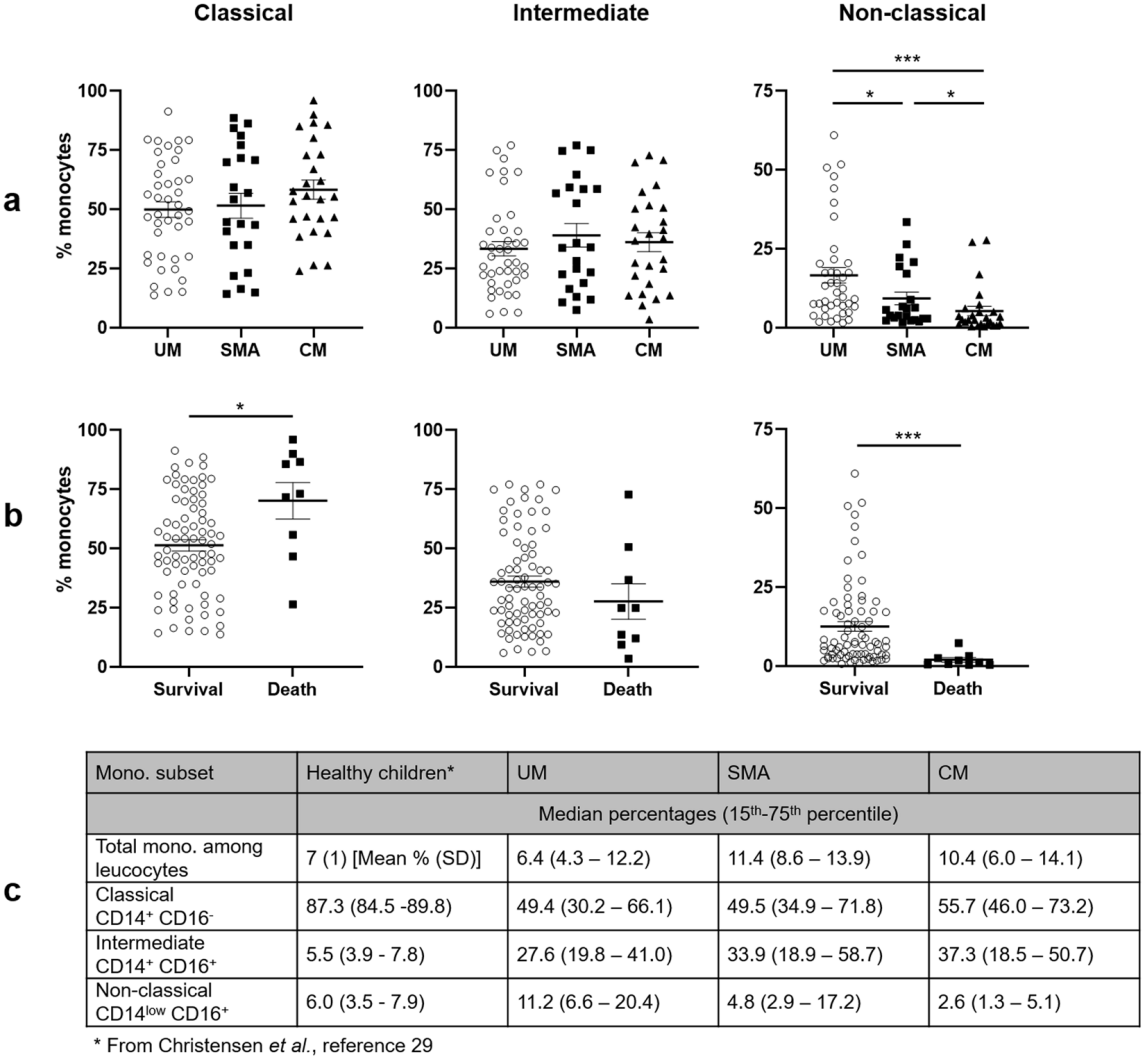
Percentages of CD14 CD16 monocyte subtypes according to malaria severity and death occurrence. Data shown are median percentages of CD14^hi^ CD16^−^, CD14^hi^ CD16^+^, and CD14^low^ CD16^+^ monocytes in the different clinical groups (**a**) and according to survival or death (**b**). Detailed values are shown for healthy children (values from Christensen *et al*., reference^29^, with a mean age of 9.9 years (±2.3) (mean ± SD), n = 20) and for our subjects (**c**). Values were compared by the Mann-Whitney U-test. *P < 0.05, **P < 0.005, ***P < 0.0005. UM: uncomplicated malaria, SMA: severe malarial anemia, CM: cerebral malaria.

To distinguish and define CD14 CD16 monocyte subsets, we used flow cytometry (Fig. 1). To ensure that our analyses were not due to cell death, we compared the percentages of live cells between clinical groups and by clinical outcome. The percentage of live cells was slightly higher in the SMA group compared to UM and CM groups (P = 0.02 and P = 0.01, respectively), but percentages were similar between survival and death groups (Fig. 1e). The percentages of monocyte subsets were compared according to the clinical group (UM, SMA or CM), and to the vital prognosis (Fig. 2). Percentages of classical and intermediate monocytes were similar in all three clinical groups (Fig. 2a). Conversely, the decreased percentage of non-classical monocytes was notably related to severity and to death occurrence (Fig. 2a,b). Higher percentages of classical CD14^+^ CD16^−^ monocytes were also related to the death outcome (Fig. 2b). Compared to healthy children^29^, the percentage of classical monocytes in the three groups of malarial subjects appeared to be much lower (Fig. 2c). Conversely, the percentage of intermediate monocytes appeared to be much higher in malaria-infected children (Fig. 2c). For non-classical monocytes, their percentage appeared higher in the UM group, similar in the SMA group and lower in the CM group, in comparison to healthy subjects, although these observations are not based on any statistical tests (Fig. 2c).

### Monocyte subtypes, plasma cytokines and parasitaemia

**Figure 3:**
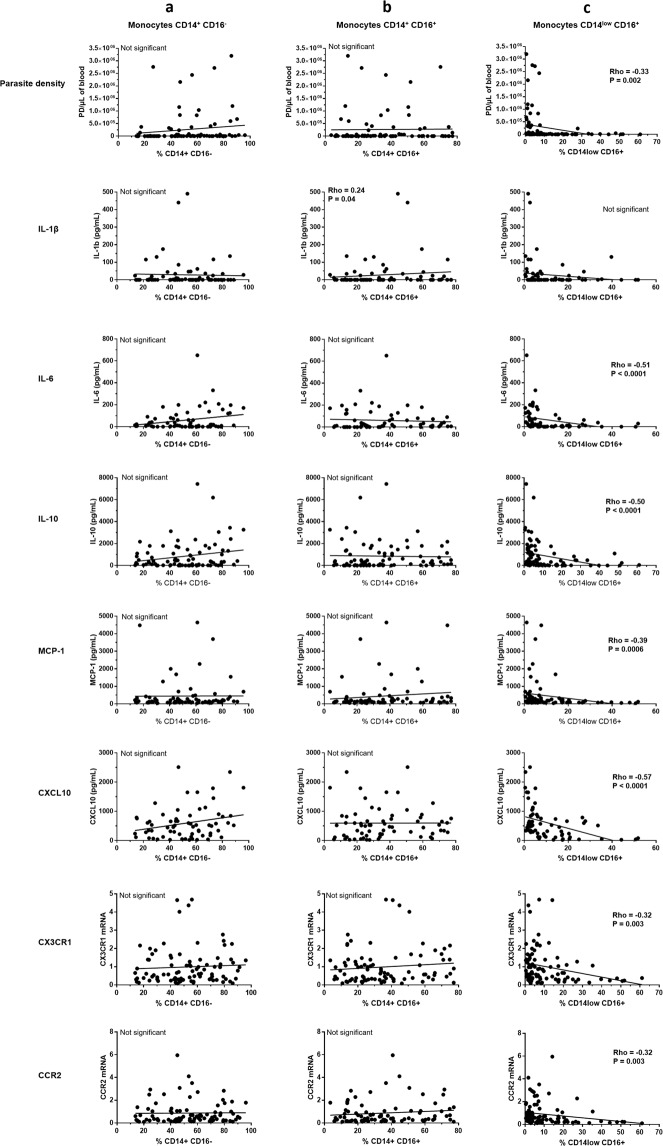
Correlations between prevalence of the three monocyte subtypes and parasite density, and plasma levels of cytokines and chemokines. Spearman correlation test was used.

As monocyte subtypes are also described by their functional capacity to produce cytokines, chemokines, to migrate and to limit microbe multiplication, we studied the potential correlations between monocyte percentages and parasite density, plasma cytokines, chemokines and chemokine receptors (Fig. 3). Interestingly, only the proportion of non-classical monocytes showed an inverse correlation with parasite density (Rho = −0.33, P = 0.002, Fig. 3c). Proportions of intermediate monocytes presented positive correlations with the pro-inflammatory cytokines IL-1β (Fig. 3b) although Rho value was quite low (<0.3). Conversely, proportions of non-classical monocytes presented strong inverse correlations with plasma IL-6, IL-10, MCP-1, CXCL10, and to a lesser extent with CX3CR1 and CCR2 mRNA (see Fig. 3c).

### Monocyte surface markers and relation to malarial severity and death

**Figure 4:**
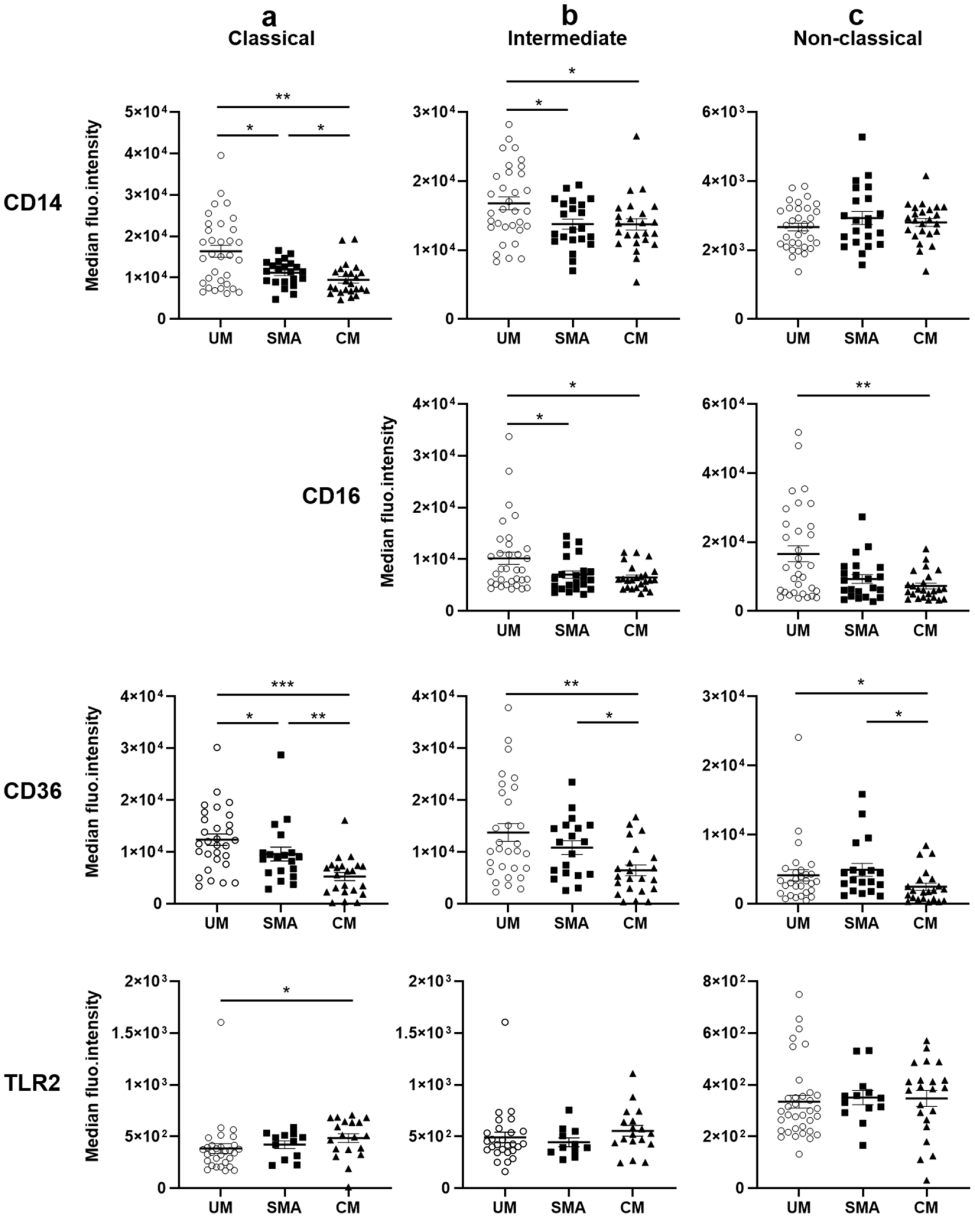
Differential protein expression of surface receptors on monocyte subsets according to severity. Data shown are median fluorescence intensities of CD14, CD16, CD36 and TLR2. (**a**) Expressed by classical monocytes (without CD16 which is not expressed in this subset), (**b**) by intermediate monocytes, (**c**) by non-classical monocytes. Values were compared by the Mann-Whitney U-test. *P < 0.05, **P < 0.005, ***P < 0.0005. UM: uncomplicated malaria, SMA: severe malarial anemia, CM: cerebral malaria.

**Figure 5:**
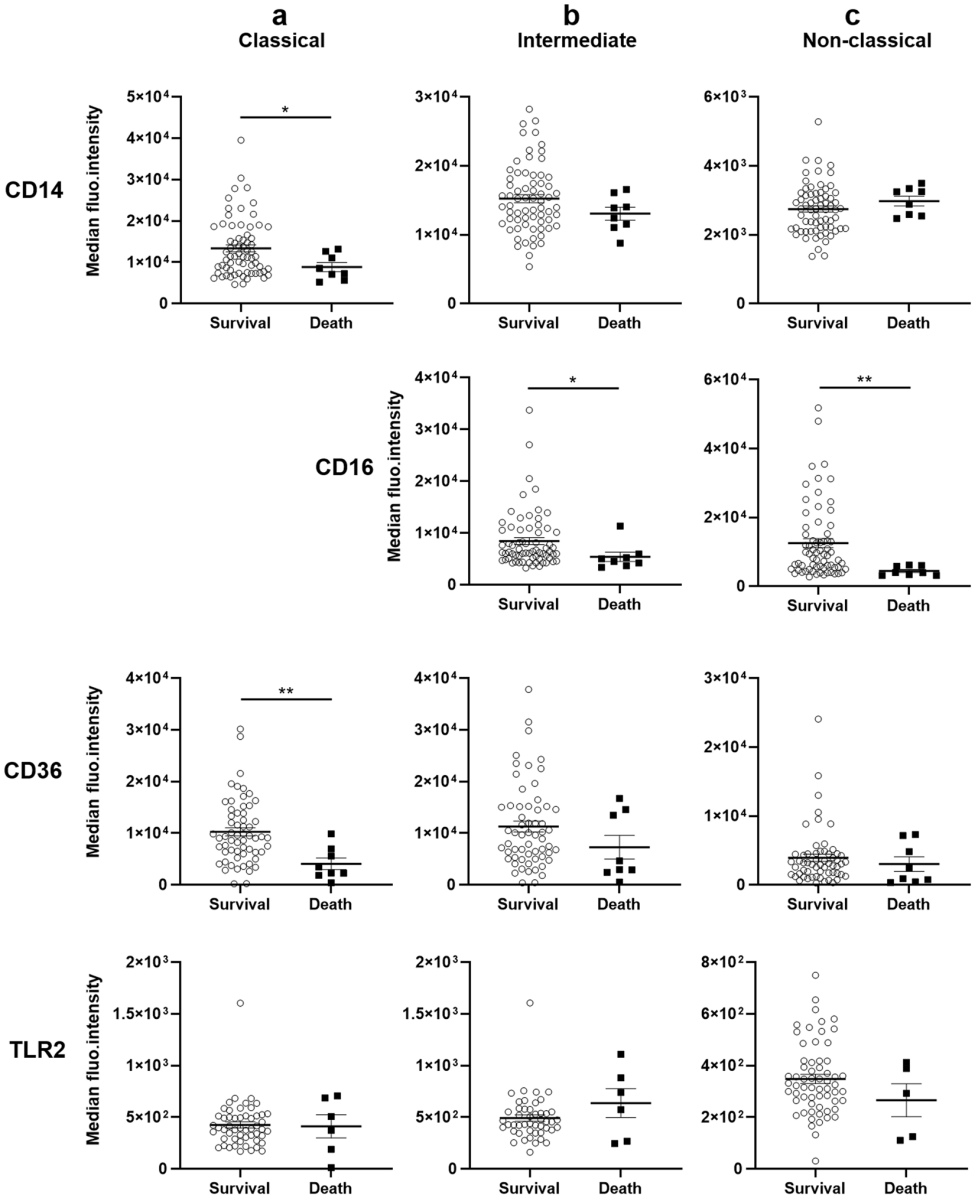
Differential protein expression of surface receptors on monocyte subsets according to survival or death. Data shown are median fluorescence intensities of CD14, CD16, CD36 and TLR2. (**a**) Expressed by classical monocytes (without CD16 which is not expressed in this subset), (**b**) by intermediate monocytes, (**c**) by non-classical monocytes. Values were compared by the Mann-Whitney U-test. *P < 0.05, **P < 0.005, ***P < 0.0005.

**Figure 6:**
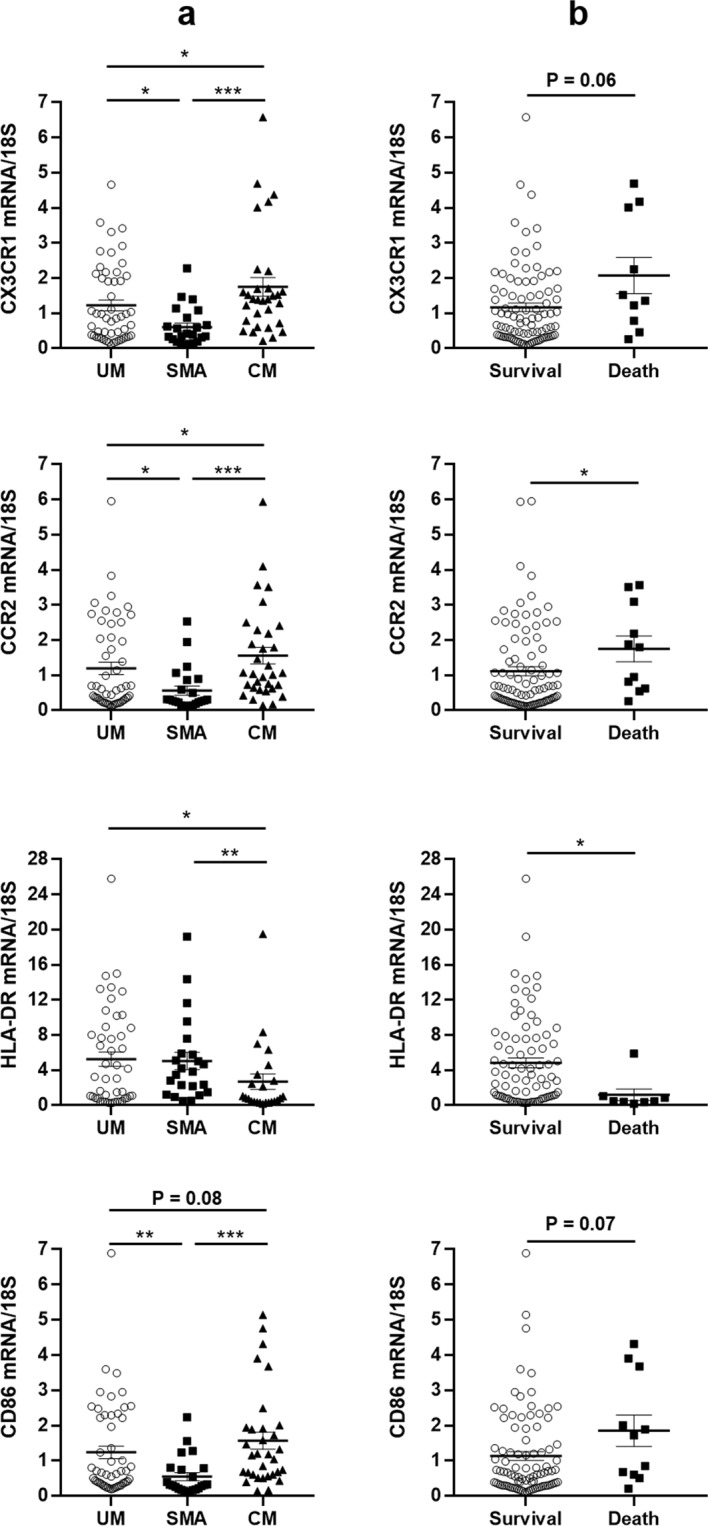
Differential gene expression of surface receptors on monocyte subsets according to severity (**a**) and fatal outcome (**b**). Values were compared by the Mann-Whitney U-test. *P < 0.05, **P < 0.005, ***P < 0.0005. UM: uncomplicated malaria, SMA: severe malarial anemia, CM: cerebral malaria.

We next studied the expression of a panel of monocyte receptors involved in various monocyte functions. Expression of CD14, CD16, CD36 and TLR2, important markers for both innate and adaptive immunity, was evaluated by flow cytometry (Table 3, Figs. 4, 5). Expression of the chemokine receptors CX3CR1 and CCR2, involved in monocyte trafficking, was measured by RT-qPCR, as well as HLA-DR and CD86 expression, two receptors implicated in T cell priming (Fig. 6). The Table 3 presents the level of expression of CD14, CD16, CD36 and TLR2 according to the monocyte subtype. Classic monocytes were characterised by high CD14 and CD36 expression in comparison to non-classical monocytes, and lower TLR2 in comparison to intermediate monocytes. Intermediate monocytes also presented high CD14 and CD36 expressions, the highest TLR2 expression, and a lower expression of CD16 in comparison to non-classical monocytes. Finally, non-classical monocytes were characterised by lower CD14, CD36 and TLR2 by higher CD16 expression (Table 3). We next studied the relationship between the expression of these receptors and severity and death. For classical monocytes, CD14 and CD36 expression was inversely related to severity, with lower MFIs in CM compared to SMA and UM (Fig. 4a). Conversely, TLR2 expression was higher in CM children than in UM children (Fig. 4a). For intermediate monocytes, CD14, CD16 and CD36 expression was also inversely related to severity with a lower expression in CM subjects compared to UM subjects (Fig. 4b). Finally, CD16 and CD36 are the two markers whose expression is related to severity for non-classical monocytes. Both were inversely related to severity with lower expression in CM subjects compared to UM subjects (Fig. 4c).

**Table 3:** Characterisation of monocyte subpopulations according to CD14, CD16, CD36 and TLR2 median fluorescence intensities (MFI).

	CD14^+^ CD16^−^ monocytes	CD14^+^ CD16^+^ monocytes	CD14^low^ CD16^+^ monocytes
CD14 MFI	11,374 (7,432–15,667)^***^	14,052 (11,680–17,465)^$$$^	2,759 (2,214–3,222)^§§§^
CD16 MFI	—	6,261 (4,961–10,564)^$$$^	7,632 (4,957–13,268)
CD36 MFI	8,630 (5,083–12,469)^*^	8,905 (4,952–14,938)^$$$^	2,954 (1,504–4,534)^§§§^
TLR2 MFI	389 (292–514)^**^	456 (369–577)^$$$^	324 (252–412)^§§^

Values presented are medians (interquartile range). Values were compared by the Wilcoxon signed-rank test and indicated by ^*^for CD14^+^ CD16^−^ versus CD14^+^ CD16^+^, ^$^for CD14^+^ CD16^+^ versus CD14^low^ CD16^+^, ^§^for CD14^+^ CD16^−^ versus CD14^low^ CD16^+^. One symbol means P < 0.05, two symbols P < 0.005 and 3 symbols P < 0.0005.

Regarding death occurrence, higher CD14 and CD36 expression by classic monocytes was associated with survival (Fig. 5a). Higher CD16 expression by both intermediate and non-classical monocytes was also related to survival (Fig. 5b,c). TLR2 expression was similar in children who died or survived regardless of the monocyte subtype (Fig. 5a–c).

Interestingly, CX3CR1 and CCR2 mRNA were overexpressed in monocytes from CM patients compared to UM and SMA (Fig. 6a), and such overexpression was also associated with death occurrence although for CX3CR1 we observed a non-significant trend (Fig. 6b). We noted that monocytes from SMA patients expressed less CX3CR1 and CCR2 than the two other groups (Fig. 6a). For CD86 mRNA expression, the profile is similar to that of CX3CR1 and CCR2, although the difference between CM and UM groups, and death and survival, is only a trend (Fig. 6a,b). Finally, monocytes from CM patients expressed less HLA-DR compared to UM and SMA monocytes (Fig. 6a) and this under-expression was associated with death occurrence (Fig. 6b).

### Logistic regression analysis for the identification of risk factors of malarial severity and death

To determine the risk factors of severity and death occurrence, we conducted two separate backward stepwise logistic regressions (Table 4). Variables directly linked to the definition of the clinical groups were excluded (coma parameters and haemoglobin level). We have chosen not to include gene expression measures (Fig. 6), as they may differ from protein expression. Variables included in the models were first selected by univariate analysis (found significant with *P* < 0.2) (Tables 1, 2 and Figs. 2, 4 and 5). Second, the resulting variables were tested for interactions between each other by Spearman correlation or Mann Whitney U-test (Supplementary Tables S1 and S2). Table 4 shows the final results of both analyses. Finally, a lower proportion of CD14^low^ CD16^+^ monocytes was found to be associated with severity when SMA and CM subjects were grouped together to define a “severe group” (OR 1.16, *P* = 0.002). The occurrence of death was strongly related to a lower proportion of CD14^low^ CD16^+^ monocytes with an odds ratio of 1.9 (*P* = 0.02).

**Table 4:** Results of the backward stepwise multinomial logistic regression analysis for the determination of risk factors of malaria severity and death. *OR: odds ratio; CI: confidence interval.

	SMA vs CM or UM			UM vs SMA or CM			Death vs Survival		
	OR*	95% CI*	*P*	OR	95% CI	*P*	OR	95% CI	*P*
CD14^low^ CD16^+^ %	0.079	0.99–1.20	0.09	**1.16**	**1.06–1.30**	**0.002**	**1.9**	**1.11–3.13**	**0.019**

## Discussion

Here, we define and quantify the monocyte subsets during acute malaria and study the main traits of monocytes associated with increased malaria severity and mortality. We show first that a lower percentage of CD14^low^ CD16^+^ monocytes was associated with more severe malaria (considering that CM is more severe than SA) and a higher risk of death; this last association was confirmed by multivariate analysis. A key role of non-classical monocytes in the evolution of diseases has rarely been reported, unlike CD14^+^ CD16^+^ intermediate monocytes. Their count consistently increases in infectious and non-infectious diseases whereas CD14^low^ CD16^+^ non-classical monocyte count is more variable, increasing in infectious diseases such as tuberculosis^30^, HIV^31^, and sepsis in neonates and young children^32^, but decreasing in dengue fever^33^ and sepsis in adult patients^34^. During non-severe *falciparum* malaria episodes, classical monocytes were predominantly reported with increased frequencies of intermediate and non-classical subpopulations^35^. In a study with SMA and CM groups, the percentage of intermediate monocytes increased in both groups when compared with the convalescent phase, but no difference was found for non-classical monocytes^36^. This study, which took place in Kenya, and ours differ mainly in the age of the patients. Kenyan children with SMA were younger (mean 18 months) than children presenting with CM (mean 31.9 months), whereas our study participants had similar ages of around 30 months. Previous studies demonstrated the impact of age on the distribution of monocyte subsets with an increase of both intermediate and non-classical monocytes in adult patients versus newborns^37^ and in young versus older adults^38^. Thus, the divergence of these results obtained in children of different ages should be interpreted with caution.

Interestingly, Dobbs *et al*. obtained results in the same direction as ours since the percentage of non-classical monocytes was lower in children with uncomplicated malaria than in healthy children of the same age^39^. In addition, a similar association was shown in patients presenting with stroke, the majority of whom had ischemic stroke, a neuro-inflammatory pathology, as is cerebral malaria^40^. The increased proportion of CD14^low^ CD16^+^ monocytes at inclusion was associated with better outcomes. In patients presenting with systemic inflammatory response syndrome, lower non-classical monocytes was also associated to death whereas intermediate and classical monocyte counts were not related to patient survival or death^41^. Urra *et al*. suggest that the non-classical monocytes are involved in tissue repair as demonstrated for LY6C^low^ monocytes in mice, a cell population similar to human CD14^low^ CD16^+^ monocytes^40^. However, a study aiming to describe functional phenotypes of the different monocyte subsets showed that non-classical monocytes display high cytotoxic and antigen presenting abilities^42^. Furthermore, they showed that non-classical monocytes were the main producers of inflammatory cytokines (TNF and IL-1β) with a strong and stable CD16 expression. Therefore, the authors propose that non-classical monocytes are pro-inflammatory cells involved in antigen presentation^42^.

In our study, a higher prevalence of non-classical monocytes was associated with lower plasma IL-6, IL-10, MCP-1, CXCL10, and lower mRNA expression of CX3CR1 and CCR2. MCP-1 and CXCL-10 are two chemokines involved in inflammatory monocytes trafficking and in Th1 response^43^. CX3CR1 (CX3CL1 receptor) and CCR2 (MCP-1 receptor) are two chemokine receptors involved in circulating monocyte recruitment to the inflammation site; CX3CL1 is also involved in monocyte attachment to the endothelium with subsequent transmigration^43^. This inverse correlation of the proportion of CD14^low^ CD16^+^ monocytes and CX3CR1 expression is surprising since this cell population has been reported as CX3CR1^high^^22^. Such a profile appears rather anti-inflammatory despite the inverse correlation with plasma IL-10, a key anti-inflammatory cytokine. In addition, higher plasma levels of IL-1β, IL-6, IL-10, CXCL10 and mRNA levels of CCR2 and CX3CR1 were associated with severity and death outcome. This suggests that pro-inflammatory response, high plasma IL-10 and increased cell trafficking are markers of poor prognosis. Since non-classic monocytes were mostly inversely correlated to these parameters, these results reinforce the idea that this monocyte subset may play an important role in resolving cerebral malaria.

In our study, CD14^low^ CD16^+^ monocytes were also defined by higher CD16 expression in comparison to CD14^+^ CD16^+^ monocytes, which is consistent with the conclusions of Mukherjee *et al*.^42^. High CD16 expression was associated with lower severity and better survival in our patients, suggesting that the role of non-classical monocytes during cerebral malaria involves the expression of CD16. The CD16 receptor is known for its critical role in immune regulation by activating the humoral response^44^, binding immune complexes leading to opsonic phagocytosis, and the release of proinflammatory cytokines that are toxic for *Plasmodium*^45^. CD16 expression may be implicated in parasite elimination, which could explain the relationship between the higher frequency of non-classical monocytes and lower parasitaemia. This monocyte subpopulation was also associated with increased vascular superoxide production in a context of vascular dysfunction^46^. A transcriptomic and proteomic study of this sub-population also showed improved aerobic respiration and ROS production capacities^47^. Such properties can also contribute to parasite elimination. However, to clarify the role of CD14^low^ CD16^+^ monocytes during malaria, additional experiments on the functionality of isolated monocyte subpopulations from patients are needed.

In our study, other parameters were also associated with severity and survival or death. Expression of CD14, CD16, CD36 and TLR2 by classical and intermediate monocytes was also related to severity and death in our study. We found that classical monocytes expressed more CD14, CD36 and less TLR2 in UM subjects in comparison to the severe groups (CM and SMA or CM alone for TLR2), and that higher expression of CD14 and CD36 by these monocytes was related to survival. The receptor CD36 is known for multiple functions such as the recognition and the elimination of apoptotic cells, a process necessary for inflammation resolution^48,49^, and parasite clearance through non-opsonic phagocytosis of iE^24,26^, which propels CD36 as a therapeutic target^27,50^. Berry *et al*. showed decreased expression of CD36 in UM patients compared with healthy subjects, and with patients who had recovered from malaria^51^. These findings were confirmed in a later study since CD36 was less expressed by classical monocytes in acute malaria patients compared to recovery and healthy subjects^39^. Our data refine these results by specifying that lower CD36 expression by both classical and intermediate monocytes is related to severity and death.

Beyond their own functions, monocytes are also key cells for the interaction and activation of T lymphocytes and humoral response via the expression of HLA and costimulatory receptors such as HLA-DR and CD86. HLA-DR is a MHC class II receptor critical for peptide presentation to CD4 T cells, while CD86 provides costimulation to T cells for activation and proliferation. They were both shown to be downregulated in SMA and CM patients compared to uninfected children and attributed to an immunosuppressive effect^52^. In our study, HLA-DR expression was lower in CM patients compared to SMA and UM and this downregulation was related to death, while CD86 expression was not clearly associated with severity or death. This result, which can be compared with that of CD16, underlines the importance of the monocyte-stimulated T response in the resolution of cerebral malaria.

In conclusion, our data provide, to the best of our knowledge, the first evidence that non-classical CD14^low^ CD16^+^ monocytes are necessary to limit death occurrence in SMA and CM forms, a result confirmed by multivariate analysis. Thus, a small proportion of non-classical monocytes may constitute a marker of poor prognosis in cerebral malaria. Further studies are needed to decipher the functions of this sub-population and understand the mechanisms involved.

## Materials and Methods

### Study site

Patients were enrolled in Cotonou (6° 21′35″ North, 2° 26′23″ East) and Sô Tchanhoué (2°24′58″ East, 6°28′09″ North). Sô-Tchanhoué is a village located in Nokoué Lake, a permanent natural lake in the southeast of Benin, 20 km north of Cotonou. In southern Benin, malaria is mesoendemic and transmission is perennial^53^. Severe malaria mainly occurs during the rainy season (April to mid-July).

### Patients and ethics statement

Ethical clearance was obtained from the Institutional Ethics Committee of the Institut des Sciences Biomédicales Appliquées in Cotonou, Benin. All study procedures were performed in accordance with the institutional policies, guidelines and regulations pertaining to research involving human subjects. Children under 5 years old were enrolled during the rainy season (May to July) in 2014 and 2016 after signed informed consent was obtained from the parents.

Severe cases (SMA and CM) were recruited from the Mother and Child University and Hospital Center (CHUMEL), Cotonou, a referral hospital for severe malaria. Patients were managed by the medical team of each health facility. Uncomplicated cases (UM) were recruited in the medical centre of Saint-Joseph in Sô Tchanhoué. Each child was clinically examined at admission. A rapid diagnostic test for *P. falciparum* (Malaria antigen P.f test, SD Bioline) and for HIV (Alere Determine HIV-1/2, Abbott) was performed, and a venous blood sample was collected for parasitaemia measurement by thick blood smear and for isolation of peripheral blood mononuclear cells (PBMC). Children who tested negative for *P. falciparum* and positive for HIV infection were excluded from the study. After blood sampling, an adequate antimalarial treatment was administered according to the national policy (oral artemether-lumefantrine combination for UM, intravenous quinine or artesunate for CM and SMA).

As proposed by the World Health Organization (WHO)^54^, SMA cases were defined as children with asexual *P. falciparum* parasitaemia, a haemoglobin (Hb) level ≤ 5 g/dl and absence of neurological signs. Cerebral malaria was defined as asexual *P. falciparum* parasitaemia and a Blantyre coma score ≤ 2 combined with a coma duration of six hours at least, with exclusion of other causes for coma^54^, particularly trauma and meningitis controlled by lumbar puncture when indicated. Children presenting UM had a positive diagnosis for *P. falciparum* infection combined with fever >37 °C and no sign of severity. A questionnaire was completed with the parents in order to collect social data and clinical signs and treatment received before hospital admission.

### Blood collection and processing

A biological assessment was carried out by the medical team at admission including haemoglobin, glycemia and blood group. For research purposes, a minimum of 4 mL peripheral venous blood was collected at admission from all patients in a vacutainer tube containing EDTA, and processed within 4 hrs. Giemsa-stained thick blood smears were prepared from venous blood collected in EDTA tubes to quantify the parasite load. Plasma was sampled after centrifugation and preserved at −80 °C. Human PBMC were isolated by a density gradient centrifugation method on Lymphoprep (Axis-Shield PoC AS, Oslo, Norway). Cells were frozen in RPMI supplemented with 30% foetal bovine serum and 30% DMSO in liquid nitrogen until cytometry experiments.

### IL-1β, IL-6, IL-10, MCP-1 and CXCL10 plasma titration

The levels of IL-1β, IL-10, and MCP-1 in plasma samples were determined using commercially available OptiEIA kits (BD Biosciences, France). IL-6 and CXCL10 plasma levels were tested using R & D Systems DuoSet (Bio-Techne, France). As suggested by the manufacturer’s instructions, we established the lower limits of detection (LLDs) for the five parameters tested, as follows: (mean negative control optical density) +2 * (Standard deviation of negative control optical density). Our LLDs were 14.7, 4.8, 22.2, 30.4 and 31.3 pg/ml for IL-1β, IL-6, IL-10, MCP-1 and CXCL10 respectively.

### Analysis of monocyte subpopulations by flow cytometry

Peripheral blood mononuclear cells were stained with fluorochrome labelled antibodies: anti-CD14 peridinin chlorophyll protein combined to Vio 700 Dye (PerCPVio700), anti-CD16 phycoerythrin (PE), anti-CD36 fluorescein isothiocyanate (FITC), anti-TLR2 allophycocyanin (APC) (all from Miltenyi Biotec, France), and live-dead-violet (Life Technologies, France) to exclude dead cells. For each sample, all cells were analysed in order to maximize the number of cells of interest. So, a minimum of 30,000 total events were acquired for each sample (median number of events acquired 166,126, 25^th^ percentile 113,351, 75^th^ percentile 233,360) resulting in a minimum of 1000 live cells of interest analysed for each sample. Acquisitions were performed on a cytometer Fortessa (BD Biosciences, France). Forward and side scattering parameters (FSC and SSC) were first used to gate on monocytes among other leucocytes. Three CD14 CD16 monocytes subsets were defined according to anti-CD14 and anti-CD16 labelling (see Fig. 1). Data analysis was carried out on DIVA software. Data are presented as the percentage of positive cells from the total gated monocytes, or as the median fluorescence intensity (MFI) of the receptor studied in the population gated.

### Monocyte isolation and mRNA expression measurements by RT-qPCR

Monocytes were isolated, RNA was extracted and CX3CR1, CCR2, HLA-DR and CD86 genes were amplified by qPCR as described before^23^. The primers used were designed using Primer 3 software, as follows: CX3CR1 sense 5′ ATA-TCC-CCT-CCC-AGT-CAC-CT, antisense 5′ GGT-CTG-GAC-GGG-TGA-ATA-CA; CCR2 sense 5′ TGG-CTG-TGT-TTG-CTT-CTG-TC, antisense 5′ CCC-GAG-TAG-CAG-ATG-ACC-AT; HLA-DR sense 5′ AGG-CAG-CAT-TGA-AGT-CAG-GT, antisense 5′ CTC-AGC-ATC-TTG-CTC-TGT-GC; CD86 sense 5′ ACA-GAT-GTC-CTA-CGG-GAA-CG, antisense 5′ ATC-CCA-CCT-TAG-AGC-CAG-GT; Housekeeping gene 18S sense 5′ AAA-CGG-CTA-CCA-CAT-CCA-AG, antisense 5′ CCT-CCA-ATG-GAT-CCT-CGT-TA.

### Statistical analysis

Statistical analysis was performed on Statview software (Abacus Concepts Berkeley, CA) and Prism software (version 8.1.0). Non parametric tests were used for all analyses. Mann-Whitney U-test was used to compare continuous values between the different clinical groups (UM, SMA, CM), after validation of significant differences between the three groups by the Kruskall-Wallis test. To study quantitative variables, the Spearman’s rank correlation test was used to assess correlations and the Wilcoxon signed-rank test to compare values. To determine the most significant parameters regarding severity and death occurrence, two multivariate analyses were then performed by logistic regression. Only variables that were found to be significant by univariate analysis with *P* < 0.2, that were not directly related to the definition of the clinical groups (i.e. seizure, Blantyre score, haemoglobin), and variables found to be independent of each other, were tested by backward stepwise logistic regression (see Supplementary Tables S1 and S2). *P* values < 0.05 were considered statistically significant.

## Supplementary information

Supplementary Tables 1 and 2
