# Supplementary material for: Changes in monocyte subsets are associated with clinical outcomes in severe malarial anaemia and cerebral malaria
Source: Sci Rep. 2019 Nov 26;9:17545. doi: 10.1038/s41598-019-52579-7 (PMC6879635; doi:10.1038/s41598-019-52579-7)
Supplement: Supplementary file 1 — Supplementary Tables 1 and 2 [file 41598_2019_52579_MOESM1_ESM.pdf]

Supplementary information

Changes in monocyte subsets are associated with clinical outcomes in severe malarial anemia and cerebral malaria

Jade Royo[1], Mouna Rahabi[1], Claire Kamaliddin[2], Sem Ezinmegnon[3], David Olagnier[4], Hélène Authier[1], Achille Massougbodji[3], Jules Alao[e], Yélé Ladipo[5], Philippe Deloron[2], Gwladys Bertin[2], Bernard Pipy[1], Agnès Coste[1], Agnès Aubouy[1*]

	**% $CD14^{low}$ $CD16^{+}$**	CD36 MFI ($CD14^{+}$ $CD16^{-}$)	**CD36 MFI ($CD14^{+}$ $CD16^{+}$)**	CD16 MFI ($CD14^{+}$ $CD16^{+}$)	CD16 MFI ($CD14^{low}$ $CD16^{+}$)	CD14 MFI ($CD14^{+}$ $CD16^{-}$)	**CD14 MFI ($CD14^{+}$ $CD16^{+}$)**	TLR2 MFI ($CD14^{+}$ $CD16^{-}$)	TLR2 MFI ($CD14^{low}$ $CD16^{+}$)	Pl. IL-1β	Pl. IL-6	Pl. IL-10	Pl. MCP-1	Pl. CXCL 10	Pr. hosp	Temp.	Parasite density
**% $CD14^{low}$ $CD16^{+}$**		0.0006	**ns**	0.0009	<0.0001	0.02	**ns**	0.04	**ns**	**ns**	<0.0001	<0.0001	0.0006	<0.0001	0.03*	0.02	0.002
CD36 MFI ($CD14^{+}$ $CD16^{-}$)			<0.0001	ns	<0.0001	0.005	ns	<0.0001	0.02	0.05	<0.0001	0.0002	0.001	<0.0001	ns*	0.03	0.002
**CD36 MFI ($CD14^{+}$ $CD16^{+}$)**				ns	ns	ns	**ns**	0.005	0.005	ns	0.02	ns	ns	ns	ns*	ns	0.006
CD16 MFI ($CD14^{+}$ $CD16^{+}$)					<0.0001	0.0002	0.003	0.03	ns	ns	ns	0.05	ns	0.01	0.05*	ns	ns
CD16 MFI ($CD14^{low}$ $CD16^{+}$)						<0.0001	0.0003	0.0001	ns	ns	ns	0.03	ns	0.0004	ns*	ns	ns
CD14 MFI ($CD14^{+}$ $CD16^{-}$)							<0.0001	0.01	ns	ns	0.001	<0.0001	0.008	0.0002	0.02*	ns	ns
**CD14 MFI ($CD14^{+}$ $CD16^{+}$)**								ns	ns	ns	0.002	<0.0001	0.05	0.002	ns*	ns	ns
TLR2 MFI ($CD14^{+}$ $CD16^{-}$)									0.02	ns	ns	ns	ns	ns	ns*	0.04	0.056
TLR2 MFI ($CD14^{low}$ $CD16^{+}$)										ns	ns	0.03	ns	0.03	ns*	ns	ns
Pl. IL-1β											<0.0001	0.0004	<0.0001	<0.0001	ns*	0.0004	<0.0001
Pl. IL-6												<0.0001	<0.0001	<0.0001	ns*	<0.0001	<0.0001
Pl. IL-10													<0.0001	<0.0001	ns*	<0.0001	<0.0001
Pl. MCP-1														<0.0001	ns*	0.008	0.006
Pl. CXCL10																<0.0001	<0.0001
Pr. hosp																ns*	ns*
Temp.																	0.008
Parasite density																	

Supplementary Table S1. Correlation matrix used to choose variables to be tested in the multivariate model of the risk of malaria severity. Interactions were tested mostly by Spearman correlation. *Interactions were tested by the Mann-Whitney U-test.

For the first multivariate model based on malaria severity, correlated variables were excluded. We began our selection by excluding variables correlating with immunologic factors. Thus, the first row leads to the selection of: % $CD14^{low}$ $CD16^{+}$, CD36 MFI (for $CD14^{+}$ $CD16^{+}$ cells), CD14 MFI (for $CD14^{+}$ $CD16^{+}$ cells), TLR2 MFI ($CD14^{low}$ $CD16^{+}$) and plasma IL-1β. We then tested interactions between these 5 variables. TLR2 MFI ($CD14^{low}$ $CD16^{+}$) and plasma IL-1β were excluded as they both interact with CD36 MFI (for $CD14^{+}$ $CD16^{+}$ cells). The variables selected were finally: % $CD14^{low}$ $CD16^{+}$, CD36 MFI (for $CD14^{+}$ $CD16^{+}$ cells) and CD14 MFI (for $CD14^{+}$ $CD16^{+}$ cells).

	**% $CD14^{low}$ $CD16^+$**	**% $CD14^+$ $CD16^+$**	% $CD14^+$ $CD16^-$	CD36 MFI ($CD14^+$ $CD16^-$)	**CD36 MFI ($CD14^+$ $CD16^+$)**	CD16 MFI ($CD14^+$ $CD16^+$)	CD16 MFI ($CD14^{low}$ $CD16^+$)	CD14 MFI ($CD14^+$ $CD16^-$)	**CD14 MFI ($CD14^{low}$ $CD16^+$)**	Pl. IL-1β	Pl. IL-6	Pl. IL-10	Pl. MCP-1	Pl. CXCL10	Prev. hosp	Temp.	PD
**% $CD14^{low}$ $CD16^+$**		**ns**	<0.0001	0.0006	**ns**	0.0009	<0.0001	0.02	**ns**	**ns**	<0.0001	<0.0001	0.0006	<0.0001	0.03*	0.02	0.002
% $CD14^+$ $CD16^+$			<0.0001	ns	**ns**	ns	ns	ns	**ns**	0.04	ns	ns	ns	ns	ns*	ns	ns
% $CD14^+$ $CD16^-$				ns	ns	0.03	0.02	ns	ns	ns	ns	ns	ns	ns	ns*	ns	ns
CD36 MFI ($CD14^+$ $CD16^-$)					<0.0001	ns	<0.0001	0.005	ns	0.05	<0.0001	0.0002	0.001	<0.0001	ns*	0.03	0.002
**CD36 MFI ($CD14^+$ $CD16^+$)**						ns	ns	ns	**ns**	ns	0.02	ns	ns	ns	ns*	ns	0.006
CD16 MFI ($CD14^+$ $CD16^+$)							<0.0001	0.0002	0.003	ns	ns	0.05	ns	0.01	0.05*	ns	ns
CD16 MFI ($CD14^{low}$ $CD16^+$)								<0.0001	0.0003	ns	ns	0.03	ns	0.0004	ns*	ns	ns
CD14 MFI ($CD14^+$ $CD16^-$)									<0.0001	ns	0.001	<0.0001	0.008	0.0002	0.02*	ns	ns
**CD14 MFI ($CD14^{low}$ $CD16^+$)**										ns	ns	ns	ns	ns	ns*	ns	0.03
Pl. IL-1β											<0.0001	0.0004	<0.0001	<0.0001	ns*	0.0004	<0.0001
Pl. IL-6												<0.0001	<0.0001	<0.0001	ns*	<0.0001	<0.0001
Pl. IL-10													<0.0001	<0.0001	ns*	<0.0001	<0.0001
Pl. MCP-1														<0.0001	ns*	0.008	0.006
Pl. CXCL10																<0.0001	<0.0001
Pr. hosp																ns*	ns*
Temp.																	0.008
PD																	

Supplementary Table S2. Correlation matrix used to choose variables to be tested in the multivariate model of the risk of malarial death Interactions were tested mostly by Spearman correlation. *Interactions tested by Mann-Whitney U-test.

For the second multivariate model based on malarial death, the strategy was similar. Thus, the first row leads to the selection of: % $CD14^{low}$ $CD16^+$, % $CD14^+$ $CD16^+$, CD36 MFI (for $CD14^+$ $CD16^+$ cells), CD14 MFI (for $CD14^{low}$ $CD16^+$ cells) and plasma IL-1β. We then tested interactions

between these 5 variables and plasma IL-1β was excluded as it interacts with % $CD14^+$ $CD16^+$. The variables selected were finally: % $CD14^{low}$ $CD16^+$, % $CD14^+$ $CD16^+$, CD36 MFI (for $CD14^+$ $CD16^+$ cells) and CD14 MFI (for $CD14^{low}$ $CD16^+$ cells).
